# Using Carrier DNA in Ultra-Low Input Library Preparations for Next-Generation Sequencing

**DOI:** 10.7171/001c.157570

**Published:** 2026-03-06

**Authors:** Ping Li, Jeremy Kahsen, Karen Olsson-Francis, Stefan J. Green

**Affiliations:** 1 Genomics and Microbiome Core Facility Rush University Medical Center; 2 AstrobiologyOU, School of Environment, Earth and Ecosystem Sciences The Open University; 3 Division of Infectious Diseases, Department of Internal Medicine Rush University Medical Center

**Keywords:** next-generation sequencing, DNA, shotgun metagenome sequencing, library preparation

## Abstract

The purpose of this study was to evaluate the use of carrier DNA (i.e., exogenous DNA spike-in) for shotgun metagenome sequencing of ultra-low levels (less than 50 picograms) of metagenomic DNA. The study hypothesized that carrier DNA would improve the robustness of library preparation for samples with DNA concentrations that are below detection by providing a tangible amount of known DNA thereby bringing total DNA concentrations closer to recommended input ranges for metagenomic library kits. The study employed adaptive polymerase chain reaction (PCR) cycling using an iconPCR instrument (N6tec) to allow dynamic thermocycling until a sufficient library for sequencing was amplified, regardless of the input DNA concentration. Libraries were sequenced and mapped in order to reference genomes of Lambda and mock community organisms, and outcome measures included total reads, on-target reads, evenness of coverage across 10 organisms within each mock community, and PCR duplication rate. The study demonstrated that libraries can be prepared down to 50 fg of input DNA, but there is a strong correlation between input DNA concentration and PCR duplication rate. The utility of spiking in carrier DNA is equivocal as it has mild negative impacts on the observed distribution of mock communities and serves as a loss of sequencing output. Although the loss of sequencing capacity due to carrier DNA can be partially offset by a reduced loss of data from PCR duplication, carrier DNA spike-in is not recommended for routine library preparation of ultra-low input samples. Adaptive cycling allows for appropriate cycling conditions when input DNA concentrations are below detection.

## Introduction

The ability to make next-generation sequencing libraries from ultra-low amounts of DNA (e.g., ≤10 pg)^1^ is important across a wide range of fields including cleanroom monitoring, extreme environmental samples such as aerosols, skin/ocular surface swabs, and others.^2,3^ In most cases, extracted DNA concentrations are below detection when using fluorometric quantification, but the sequence data are still desired. Prior studies with mock DNA templates diluted to sub-pg levels show that direct library preparation can be preferable to whole genome amplification.^3^ However, the lack of measurable DNA can be challenging for library preparation due to uncertainty in the number of PCR cycles for library amplification. The use of carrier DNA in the metagenomic sequencing of very low biomass specimens has been previously explored in a limited fashion, including in stable isotope probing metagenomic experiments^4^ as well as in other low biomass systems and for Oxford Nanopore Technologies sequencing (e.g., CarrierSeq).^5^ In such systems, the carrier DNA is meant to bring the sample into DNA concentration ranges recommended by library preparation kits or enable the recovery of sufficient quantities of labeled DNA for metagenomics.^4^

This study aimed to develop best practice strategies for ultra-low biomass shotgun metagenomics using two mechanisms: the use of a carrier DNA spike-in of Lambda DNA and the use of an adaptive PCR cycling instrument (iconPCR, N6tec)^6^ to allow dynamic thermocycling until a sufficient library was amplified. Adaptive PCR cycling, as performed by the iconPCR instrument, uses real-time fluorescence data from individual reactions to determine how many PCR cycles should be performed for each sample. In the simplest form of adaptive cycling, a fluorescence threshold is set, and each sample is cycled until that threshold is achieved. This is achieved by having independent control for every well in a 96-well thermocycler. For ultra-low input samples, adaptive cycling can be beneficial by allowing samples to cycle until an appropriate amount of library has been generated, especially in cases where input sample concentrations are not known a priori. This contrasts with standard library preparation protocols that have recommended cycle numbers for measurable DNA inputs.

To examine these two mechanisms, mock community DNA samples and spike-in DNA samples were sheared and mixed in various ratios. These combined DNAs were diluted across a range of very low input concentrations and used as input for library preparation, followed by amplification with an iconPCR device. Subsequently, the libraries were pooled and sequenced. Outcome measures included the number of reads, percentage of on-target mapping reads, ratio of spike-in-to-mock DNA template reads, the recovery of the expected ratio of mock organisms, and the rate of PCR duplicate generation. The results indicate that sample libraries can be made from ultra-low DNA inputs using this approach with viable performance metrics at 500 fg and poor, but still detectable, performance at 50 fg input levels. DNA spike-ins had very modest negative impacts on the recovery of mock community profiles. Adaptive PCR cycling allowed for simultaneous amplification of libraries across four orders of magnitude with the cycle number impacting the total library yield.

## Materials and Methods

### DNA Templates

Three DNA templates were used for analysis in this study, including purified *Enterobacteria* phage Lambda DNA (Lambda DNA; Oxford Nanopore Technologies, Oxford, United Kingdom, EXP-CTL001), ZymoBIOMICS Microbial Community DNA Standard (Zymo Research, Tustin, CA, United States, D6306), and Mycobiome Genomic DNA Mix (American Type Culture Collection (ATCC), MSA-1010). Prior to dilution, DNA concentrations were measured using Qubit fluorimetry (Thermo Fisher Scientific, Waltham, MA, United States). DNA samples were then sheared using the RapidShear™ 24-in-5 gDNA Shearing System (Triangle Biotechnology) following the manufacturer’s protocol. Briefly, 45 µL of DNA at 2–10 ng/µL was mixed with 5 µL of RapidShear reagent and sonicated for 6 minutes. The resulting average fragment size ranged from 450 to 550 base pairs. DNA templates were then mixed in varying proportions, including 100% Zymo mock DNA (Z), 100% Lambda DNA (L), 1:1 Lambda-Zymo DNA (L1Z1), 3:1 Lambda-Zymo DNA (L3Z1), 5:1 Lambda-Zymo DNA (L5Z1), 10:1 Lambda-Zymo DNA (L10Z1), 1:1 Lambda-ATCC Mycobiome DNA (1LA1), and no-template controls (NTCs). After combining sheared template and sheared Lambda DNA, samples were diluted across multiple orders of magnitude, including 5 pg/µl, 500 fg/µl, 50 fg/µl, and 5 fg/µl of total DNA. Template mixing, shearing, and dilution were performed independently twice in workflows conducted approximately 1 month apart. In the first workflow, dilutions were performed to achieve a minimum amount of 500 fg per reaction. In the second workflow, the lowest dilution contained 50 fg per reaction.

### Library Preparation

All reactions were performed in technical triplicate using a ThruPLEX DNA-Seq Kit (Takara Bio USA, Inc., San Jose, CA, United States; catalog #R400676) with unique dual indexing according to the manufacturer’s instructions with modifications during final PCR cycling. Maximum volumes of 10 µl of sheared DNA were employed, leading to total input DNA amounts of 50 pg, 5 pg, 500 fg, 50 fg, and no input (NTCs). The minimum input amount recommended by the standard protocol is 50 pg. Library preparation is a single-tube procedure including DNA repair, stem-loop adapter blunt-end ligation, extension of templates, and cleavage of loops. The final adapter-ligated templates are amplified with unique dual indexing primers. Library amplification reactions included 0.5 µL of 200x SYBR Green I dye per 100 µL (1X final working concentration), and cycling was initiated on a C1000 Touch Thermal Cycler (Bio-Rad, Hercules, CA, United States) using the following conditions: (a) Extension and cleavage: 72°C for 3 minutes and 85 °C for 2 minutes, respectively, and denaturation: 98°C for 2 minutes; (b) index incorporation: four cycles of 98°C for 20 seconds, 67°C for 20 seconds, and 72°C for 40 seconds; and (c) library amplification (recommended 6–16 additional cycles) with cycling conditions of 98°C for 20 seconds and 72°C for 50 seconds. This last step of library amplification was performed on the iconPCR platform (n6 Tec) with real-time, fluorescence-based normalization. The selected auto-normalization method chosen was “target fluorescence,” set at 10,000 relative fluorescence units, with a maximum of 20 additional PCR cycles allowed. Standard kit recommendations from Takara indicate 15–16 cycles (library amplification stage) for 50 pg inputs. Library pools were purified using KAPA Pure Beads (Roche, Basel, Switzerland), quantified using a Qubit4 Fluorometer with the Qubit dsDNA HS Assay Kit (Thermo Fisher Scientific), and assessed for fragment size distribution on a TapeStation device using D1000 ScreenTape (Agilent Technologies, Santa Clara, CA, United States).

### Sequencing

Libraries were pooled in equal volume for each sequencing run without any further attempt at library balancing. Therefore, within each experiment, read depth was an outcome measure. Library pools were sequenced on an Illumina MiniSeq platform using a paired-end 151 × 8 × 8 × 151 run configuration with a 5% PhiX control spike-in. Raw sequences from two independent sequencing runs were deposited in the Sequence Read Archive (SRA) under the Bioproject accession PRJNA1275972. Sample processing, library preparation, and sequencing were performed in the Genomics and Microbiome Core Facility at Rush University Medical Center.

### Contaminant Identification

Although the primary approach for analyzing sequence data was through a mapping procedure against known targets (see the following “Data Analysis” section), non-targeted annotation pipelines were employed to identify microbial DNA present as reagent contaminants. Once identified, whole genome sequences for these organisms were obtained and used as references in the mapping pipeline together with expected targets (i.e., mock community genomes and Lambda DNA). Briefly, taxonomic classification of sequence reads generated from reagent negative controls was conducted using the Kraken2 tool, a k-mer-based taxonomic classification system.^7^ The controls were analyzed using Kraken2 with a combination of reference sequence databases, including human, bacteria, viruses, fungi, and archaea. In addition, short read sequence taxonomic annotation was also performed using the software package MetaPhlAn3 (v4.0.1).^8^ Negative control taxonomic classification was performed by the Rush Research Bioinformatics Core at Rush University.

### Data Analysis

Raw sequence data were processed within the software package CLC Genomics Workbench (v25.0.1; Qiagen, Hilden, Germany). Data were initially trimmed to Q20 and mapped against mock community genomes as indicated by Zymo, ATCC, and potential contaminant organism genomes (GenBank References: CP053915, CP097636, CP016210, CP069300, CP170561, CP171806, CP078069, CP077308, CP065037, CP050454, CP000555, CP002095, CP041150, LT629971, CP116346, CP119083, CP016022, CP050124, CP192575, CP124551, CP010836, CP166581, CP046508) using default mapping parameters (length fraction of 0.5 and similarity fraction of 0.8). Outcome measures included the number of reads after quality trimming, number of mapping reads, number of reads mapping to expected references, ratio of mock community to Lambda DNA reads, and percentage of reads mapping to nonexpected references (i.e., contaminants). In addition, a univariate metric (ideal score)^9^ was calculated for each sample to evaluate how well sequencing recovered the expected distribution of microbial taxa within the mock community DNA. The ideal score was initially developed to produce a univariate metric for mock communities with an even distribution of taxa; it was later revised to accommodate mock communities with uneven distribution.^10^ Briefly, the metric is derived from Bray-Curtis dissimilarity calculations^11^ and is a summation of the absolute difference of the expected relative abundance of each taxon and the observed relative abundance of the same taxon. A value of 0 is a perfect match between expectation and observation across the entire community, while a value of 200 is the maximum value and represents complete divergence between expectation and observation. Duplicate read levels were evaluated on read mapping to Lambda DNA due to the greatest depth of coverage for this template relative to mock community genomes. Briefly, reads were mapped using default settings as previously described. Subsequently, the “Remove Duplicate Mapped Reads” algorithm was employed within CLC Genomics. Percent duplicate levels were obtained for all samples containing at least 10,000 mapping reads to the Lambda reference genome.

### Visualization

Raw counts and weighted mappings were imported into R, wrangled using the tidyverse, visualized with ggplot2, and integrated into composite figures using patchwork.^12–15^ Statistical differences between pairs were evaluated with t-tests, and group-level effects were analyzed with ANOVA, followed by Tukey’s post hoc tests when applicable.

## Results

**Figure 1.**
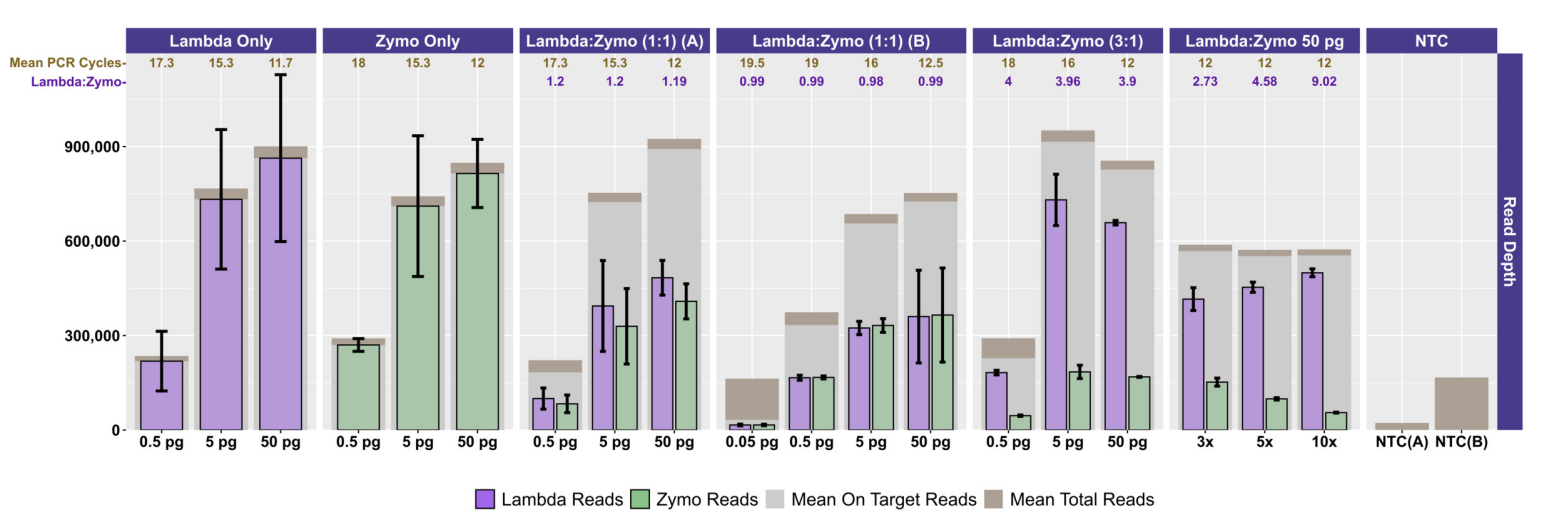
Sequencing and Mapping Metrics for Zymo Mock Community and Lambda DNA Experiments. For each experiment (Lambda DNA only; Zymo DNA only; Lambda:Zymo in 1:1 input ratio – run separately twice; Lambda:Zymo in a 3:1 ratio; Lambda:Zymo in 3:1, 5:1, and 10:1 ratios; and reagent negatives from two separate runs of NTC-A and NTC-B), total read depth is shown (brown columns) together with on-target reads (i.e., mapping to Lambda and Zymo genome sequences) in gray. For each experiment, the number of reads mapping to Lambda DNA (purple) and Zymo DNA (green) references are shown. Error bars indicate standard deviation associated with three replicates, except for two experiments where one replicate was dropped due to failed amplification. The average number of cycles of PCR used for amplification is shown at the top in brown numbering. The average measured mapping ratio of Lambda DNA to Zymo DNA is shown below the PCR cycle number in purple lettering. For most experiments, the DNA input is shown below (ranging from 0.05 to 50 pg). For the Lambda:Zymo DNA experiment comparing ratios, 50 pg inputs were used while changing the input ratio from 3:1 to 10:1.

**Figure 2.**
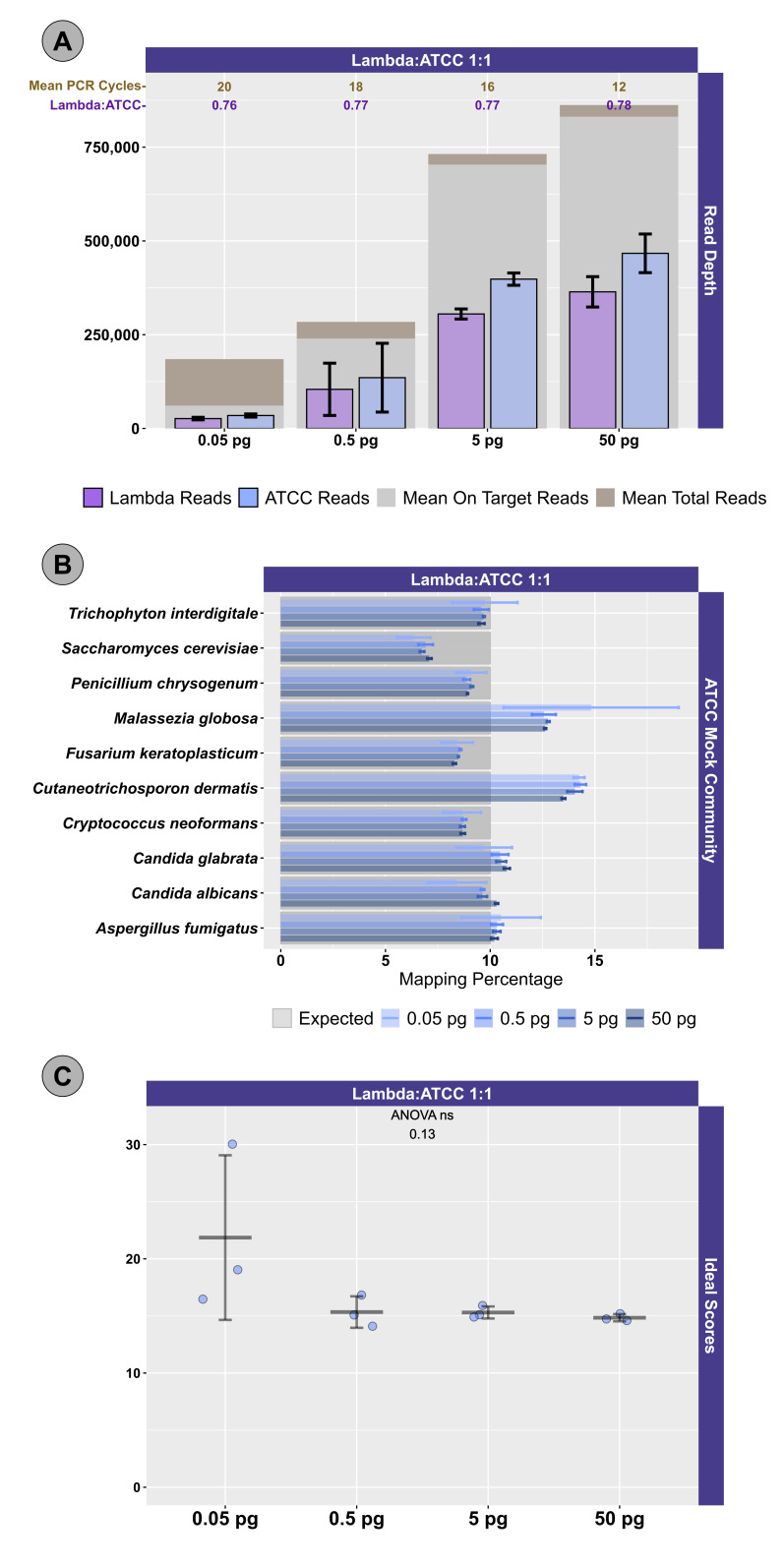
Sequencing and Mapping Metrics for ATCC Mock Community and Lambda DNA Experiment. Lambda DNA and ATCC mock community DNA were sheared, mixed in a 1:1 ratio, and diluted to yield 0.05–50 pg inputs. (A) For each input level, total read depth is shown (brown columns) together with on-target reads (mapping to Lambda and ATCC genome sequences) in gray. The number of reads mapping to Lambda DNA (purple) and ATCC DNA references (blue) are also shown. Error bars indicate standard deviation associated with three replicates. The average number of cycles of PCR used for amplification is shown at the top in brown numbering. The average measured mapping ratio of Lambda DNA to ATCC DNA is shown below the PCR cycle number in purple lettering. (B) The relative abundance of reads mapping to each of the 10 ATCC mock community fungal genomes are shown. Results are shown for libraries with 0.05–50 pg DNA input amounts. The dark gray boxes indicate the expected relative abundance of the input DNA (i.e., 10% relative abundance for each organism). Error bars indicate the standard deviation associated with three technical replicates. (C) Distribution of “ideal scores” for ATCC mock community sequences across experiments. Ideal scores are a univariate metric measuring the summed divergence of observed microbial communities from expected microbial communities. The metric ranges from 0 (perfect fit) to 200, with lower numbers indicating better matching between observed and expected relative abundances across all taxa. An ANOVA was performed to determine whether input DNA concentrations affected the observed microbial community structure. Error bars indicate the standard deviation associated with three replicates.

Two different mock community DNA samples were separately mixed with different amounts of spike-in DNA (Lambda), and libraries were prepared at DNA input levels from 50 pg to 50 fg using adaptive cycling for amplification. In the first series of experiments, only the Zymo mock community was employed, and the minimum DNA input was 500 fg. In the second series of experiments, both Zymo and ATCC mock communities were employed, and the minimum DNA input was 50 fg. Average read depth, average reads mapping to mock community DNA, average reads mapping to Lambda DNA, average adaptive PCR cycle number, and average on-target reads (i.e., reads mapping to mock DNA and Lambda DNA combined) are shown for experiments with Zymo mock community DNA in Figure 1 and ATCC mock community DNA in Figure 2A. With decreasing input, adaptive cycling increased the average number of amplification cycles. In reactions with 50 fg (0.05 pg) DNA input, cycle number reached the maximum cycles allowed (20 cycles with one exception of 19 cycles), and this was also associated with fewer total reads from 50 fg reactions. At 500 fg, a range of 18–19 cycles (with one exception with 17 cycles) was observed across all experiments, while 5 pg inputs were cycled from 16–17 cycles and 50 pg inputs were cycled from 12–13 cycles. Blanks were amplified with 19 (1 reaction) and 20 (5 reactions) cycles (Figure 1; Figure 2A).

Total sequencing depth was fairly consistent for samples with 5–50 pg inputs (534,986–~1,095,396 reads/sample with a mean of 767,600 reads), while sequencing depth decreased substantially at 500 fg (113,586–482,702 reads/sample with a mean of 282,981 reads) and 50 fg (126,844–216,212 reads/sample with a mean of 176,198 reads) despite greater PCR cycle numbers. Rates of on-target mapping were highest at 5 and 50 pg inputs (range from 95.28% to 96.77%; mean of 96.19%), slightly lower at 500 fg inputs (range from 75.43% to 93.63%; mean of 86.61%), and lowest at 50 fg inputs (range from 19.66% to 42.64%; mean of 28.45%) (Figures 1 and 2A). Negative controls (i.e., library reaction blanks) were sequenced as well, generating from 15,216 to 31,930 reads/sample (mean of 22,683) in the first experiment and a range from 98,074 to 256,896 reads/sample (mean of 166,935) reads in the second experiment (Figure 1).

We evaluated the ratio of Lambda (spike-in) to mock community sequence data against expected yields based on input DNA mixtures. In the first experiment, Lambda:Zymo DNA ratios were somewhat elevated relative to the expected ratio (Figure 1). For example, in the dilution series from 50 pg to 500 fg, Lambda:Zymo DNAs were mixed in an equimolar ratio while sequence output demonstrated elevated Lambda:Zymo (~1.2x compared to the expected 1x; Figure 1, Experiment A). Similarly, for the dilution series in the first experiment with Lambda:Zymo mixtures with 3x, more Lambda DNA, relative to Zymo DNA, sequence output ratios were elevated (~3.9–4x compared to the expected 3x). Conversely, in the second experiment (Figure 1, Experiment B), the situation was reversed with Lambda:Zymo mixtures slightly lower than expected (~0.97–1.0x compared to the expected 1x). In the second experiment, when adding Lambda DNA at increasing levels (i.e., 3x, 5x, and 10x higher than Zymo), sequence yields returned slightly lower than expected ratios (2.73x, 4.58x, and 9.02x, respectively). Similarly, when Lambda DNA was mixed with the ATCC mycobiome mock DNA at 1:1 ratios, sequence yields returned slightly lower than expected ratios (0.75–0.78x compared to the expected 1x) across all input concentrations ranging from 50 fg to 50 pg (Figure 1).

**Figure 3.**
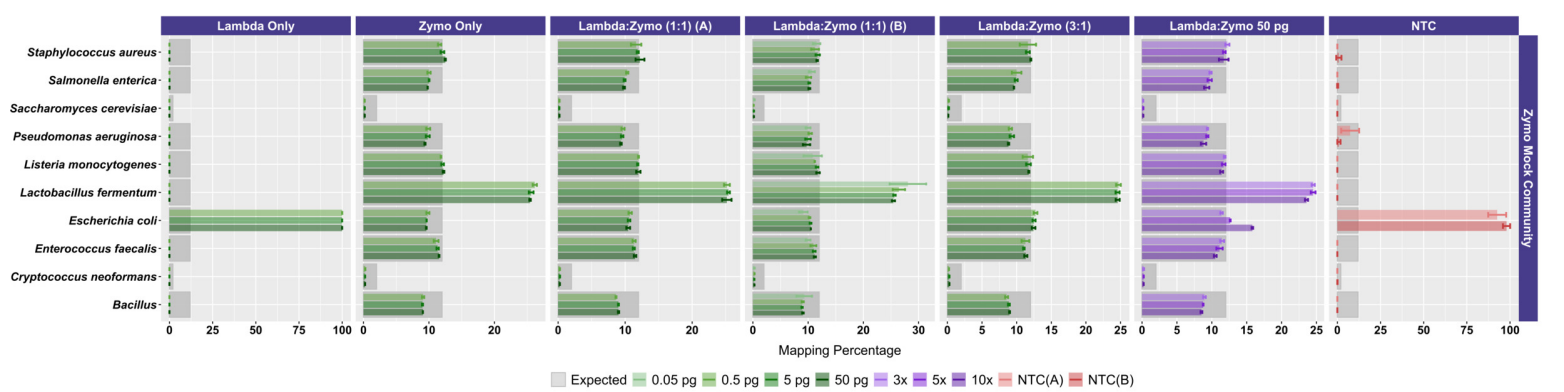
Mapping Metrics for Zymo Mock Community Sequences Across Experiments. The relative abundance of reads mapping to each of the 10 mock community microbial genomes are shown. The dark gray boxes indicate the expected relative abundance of the input DNA (i.e., 12% relative abundance for each of the eight bacterial strains and 2% relative abundance for two fungal strains). Error bars indicate the standard deviation associated with three technical replicates (most experiments). Control data, including Lambda DNA only and NTC, contain relatively few reads mapping to Zymo references with most reads mapping to the Zymo *E. coli* reference. The x-axis represents the relative abundance of reads mapping to each of the 10 reference genomes with the scale varying for each experiment.

We next examined the distribution of reads across the expected 10 microorganisms in the ATCC (Figure 2B) and Zymo (Figure 3) standards. For the ATCC standards, highly similar profiles were observed across all experiments, regardless of input DNA concentrations or ratio of Lambda DNA (Figure 2B). Fungal DNA sequences from the ATCC were detected in ratios close to even across 10 taxa; however, two fungal taxa were somewhat enriched: *Malassezia globosa* and *Cutaneotrichosporon dermatis* (Figure 2B, 2C). A slightly worse performance in recovering the expected 10% output per organism was observed at the 50 fg input level, particularly with respect to *M. globose* (Figure 2B). In experiments with Zymo standards, very poor recovery (an average of ~1/10 of expected output; Figure 3) of the two fungal taxa was observed (i.e., *Saccharomyces cerevisiae* and *Cryptococcus neoformans*), regardless of condition. Conversely, substantially higher than expected reads were mapped to *Lactobacillus fermentum* (~2.5X of expected output; Figure 3), regardless of condition. Reads mapping to *Escherichia coli* were slightly affected by the ratio of Lambda DNA, with the highest (and greater than expected) mapping reads observed with the highest (10x higher) Lambda:Zymo ratios (Figure 3). Negative controls mapped poorly to the reference genomes (a range of 40 to 151 reads/sample; mean of ~81 reads), with most reads mapping to the *E. coli* reference.

**Figure 4.**
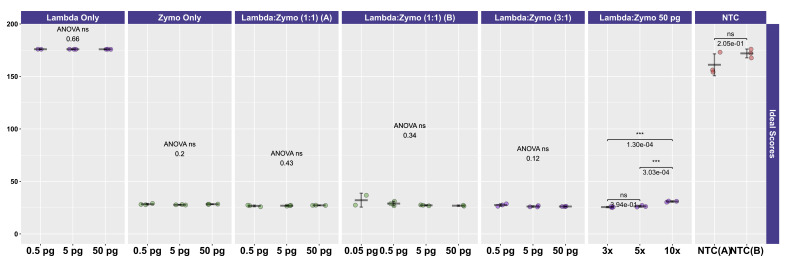
Distribution of Ideal Scores for Mapping of Sequences across Zymo Mock Community Experiments. The y-axis represents ideal scores ranging from 0 (perfect fit) to 200, with lower numbers indicating better matching between observed mapping and expected relative abundances across all taxa. Within each experiment, an ANOVA was performed to determine whether input DNA concentrations affected the observed microbial community structure (most experiments) or if the ratio of Lambda:Zymo DNA affected the observed microbial community structure. Error bars indicate that the standard deviation was associated with three replicates (most experiments). Samples without mock community DNA (Lambda only, NTCs) have high ideal scores due to mapping of a small number of reads to mock community genomes, particularly *E. coli*.

To more easily compare the conditions and mock DNAs, we used a univariate metric, the ideal score, to calculate a distance between the observed distribution of reads across the 10 reference organisms and the expected distribution of reads based on DNA ratios provided by Zymo (Figure 4) and ATCC (Figure 2C). No significant differences in ideal scores were observed frome the different input DNA concentrations, including experiments with 50 fg inputs. A slight increase in the ideal score, but not significantly different, was observed for the 50 fg input of a sample with a 1:1 Lambda:ATCC input DNA relative to 500 fg, 5 pg, and 50 pg samples (Figure 2C). Samples containing Zymo DNA had ideal scores ranging from 25.02 to 36.81 (mean of 27.61). Samples containing ATCC DNA had ideal scores ranging from 14.11 to 30.05 (mean of 16.84). Ideal scores for Zymo samples were significantly higher (p<0.001, two-tailed TTEST) relative to ATCC samples due to the poor performance of the two fungal taxa, and the overperformance of *L. fermentum*. Negative control samples had exceedingly high ideal scores as most mapping reads mapped to the *E. coli* reference genomes from the Zymo mock community. Samples with 10:1 ratios of Lambda:Zymo had slightly but significantly higher than average ideal score relative to 5:1 and 3:1 ratio samples (Figure 4).

**Figure 5.**
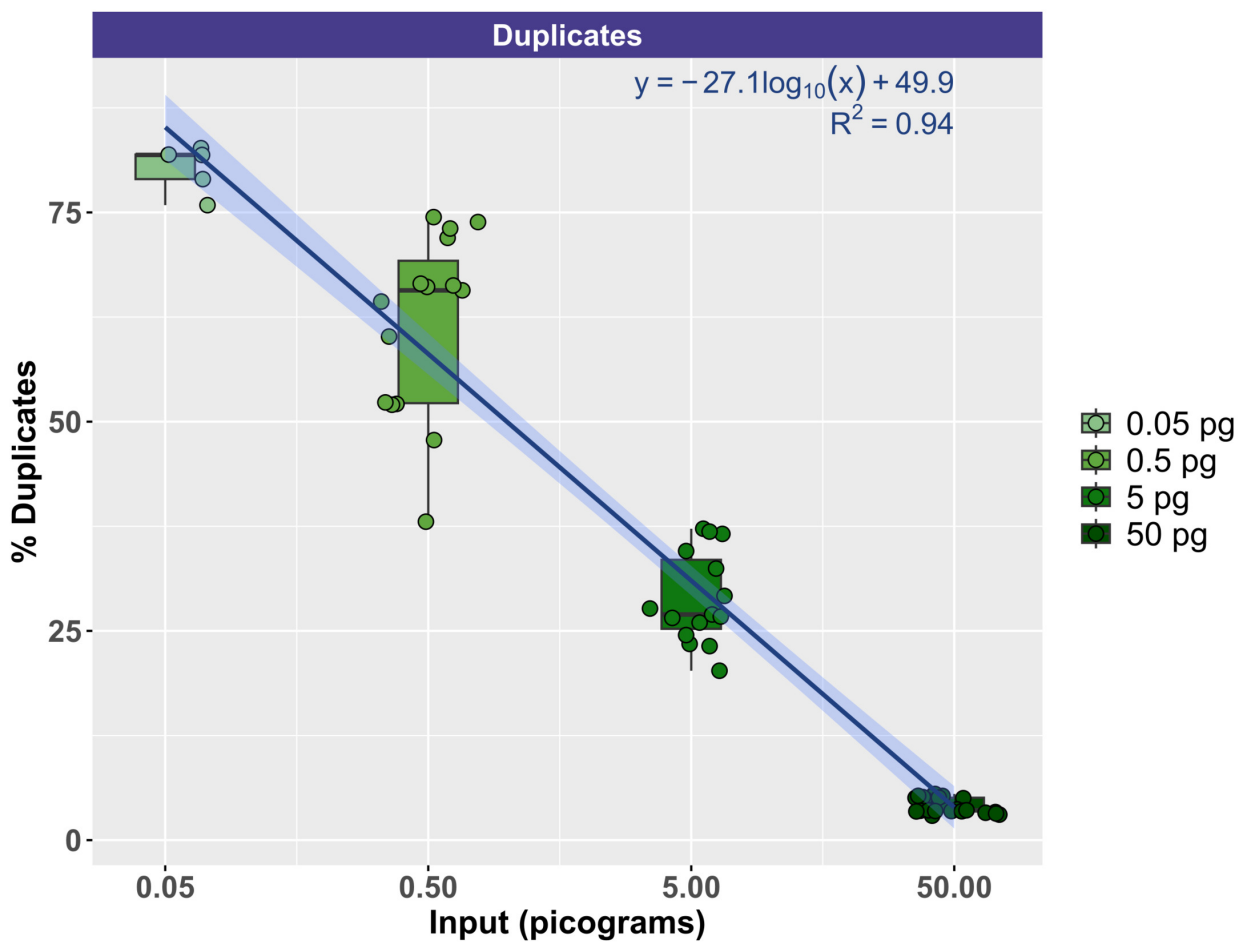
Correlation of Input DNA Levels with PCR Duplication Rates of Reads Mapping to the Lambda Genome. A strong negative correlation between DNA input levels (x-axis; pg) and PCR duplication rate (y-axis, %) was observed. This correlation fits with a logarithmic curve where y = -27.1 * log(x) + 49.9.

To evaluate the effect of input DNA concentration on library duplication, trimmed sequence reads were mapped to the Lambda genome by itself. Due to the small size of the genome, deeper sequencing was generally acquired for Lambda relative to mock community DNA, which contained 10 bacterial and fungal genomes (Zymo) or 10 fungal genomes (ATCC). Thus, the Lambda genome resequencing was more suitable for duplication examination. Figure 5 demonstrates the relationship between input genomic DNA and the duplication rate with a clear negative correlation between input DNA amount and the duplication rate. At the highest input levels (50 pg), duplication rates ranged from a minimum of 2.92% to a maximum of 5.52% (mean of 4.10%). Conversely, at the lowest input levels (50 fg), duplication rates ranged from a minimum of 75.87% to 82.68% (mean of 80.26%). The duplication rate by input DNA concentration was modeled logarithmically (Duplication rate = -27.1 * log(DNA concentration, pg) + 49.9), with strong support (R^2^ = 0.94).

## Discussion

The primary objective of this study was to determine whether nonmicrobial carrier DNA, such as Lambda DNA, was useful for generating shotgun metagenome sequence libraries from ultra-low DNA (<50 pg) samples. We hypothesized that carrier DNA could improve the library preparation for samples with DNA concentrations below detection by providing a tangible amount of known DNA and bringing total DNA concentrations into recommended ranges of low input library preparation reagents. Secondary objectives of this study were to evaluate whether adaptive PCR cycling, as performed by the iconPCR instrument (N6tec), could be integrated into library preparation workflows for samples with DNA concentrations below detection. The results in this study demonstrate that carrier DNA is not necessary from a workflow perspective when DNA concentrations are at or above 500 fg. Below 500 fg (i.e., 50 fg), sequencing performance metrics worsened substantially, which lead to fewer total reads and low on-target percentages. This is unsurprising as at 50 fg inputs, stochastic effects based on the relatively low number of target molecules become more prominent as does the impact of reagent contaminant DNA. In addition, the reduced number of template molecules also leads to a very high duplication rate. Thus, carrier DNA, on the scale of 1 pg, might be favored for samples where DNA levels are below detection and when a guaranteed library, regardless of input, is preferred. More broadly, however, larger amounts of carrier DNA led to slightly worsening performance in terms of recovering mock community profiles, but the primary concern with carrier DNA spike-in is the reduced sample sequence output due to the sequence yield from carrier DNA. However, the loss of the sample-derived sequence yield due to the presence of carrier DNA must be considered in context of the loss of unique sample-derived sequence data due to high duplication levels observed at low input levels in the absence of carrier DNA. The total yield of nonduplicated sequence data from the sample is the primary concern in any such consideration. Conversely, sequencing performance, based on mapping reads and evaluated by ideal score calculations, was shown to be similar across four orders of magnitude of input DNA and was minimally affected by carrier DNA.

The adaptive cycling of the iconPCR provides an ideal mechanism to tailor amplification strategies to input template concentrations even when below the detection of high-sensitivity fluorometric input DNA concentration measurements. Additional efforts should be taken to reduce the number of PCR cycles even further using the iconPCR to decrease the negative impact of PCR duplicate production. However, a higher maximum number of allowed cycles for amplification may be desirable when extremely low inputs are possible; in our study, 20 cycles was the maximum and with the 50 fg input led to fewer total reads relative to other input amounts. Given that an average of 17–19 cycles was observed for 500 fg input, 20 cycles was likely too few for 50 fg input amounts.

Overall, the success of this protocol should be taken within context of the manufacturer’s suggested minimum input being 50 pg, leading to library preparation from input DNA levels two-to-three orders of magnitude lower. Similar success in library preparation below manufacturer recommended inputs has been previously observed,^16^ though at higher inputs than what is described in this study. Elsewhere, library preparation from ultra-low inputs (sub-pg) has been previously demonstrated using Illumina Nextera library preparation,^17^ with levels of duplication broadly similar to those observed here. One key advantage of the workflow in this study is the use of the dynamic cycling of the iconPCR, which allows for calibrated amplification when DNA input levels are not known. Given the robustness of the overall workflow, we recommend this as a default protocol for processing ultra-low biomass specimens from spacecraft assembly facilities and other cleanrooms, aerosol samples, deep subsurface samples, and more. Furthermore, the viability of this methodology for ultra-low DNA inputs may obviate the need for whole genome amplification of ultra-low biomass samples, which can lead to substantial distortion or amplification bias of the underlying microbial community.^18,19^ Further work will examine the use of iconPCR and carrier DNA in long-read sequencing applications, such as Oxford Nanopore,^20^ where higher minimum DNA inputs are required. Limitations of the study include the use of mapping to identify on-target sequences, an approach which will not be possible for environmental samples.

### Financial Support/Conflict of Interest Disclosures

The authors declare no competing financial interests.

### Human Subjects Statement

Not applicable.

### Study Approval Statement

Not applicable.
